# Positive Delphian node in laryngeal cancer: Predictive of thyroid gland metastasis?

**DOI:** 10.1016/j.ijscr.2023.108736

**Published:** 2023-08-29

**Authors:** Barbara Verro, Antonio Lo Casto, Carmelo Saraniti

**Affiliations:** a Division of Otolaryngology, Department of Biomedicine, Neuroscience and Advanced Diagnostic, University of Palermo, 90127 Palermo, Italy; b Division of Radiology, Department of Biomedicine, Neuroscience and Advanced Diagnostic, University of Palermo, 90127 Palermo, Italy

**Keywords:** Case report, Laryngeal cancer, Delphian node, Thyroid metastasis, Head and neck cancer, Radiology

## Abstract

**Introduction:**

Laryngeal carcinoma represents the 22nd most common cancer worldwide. Thyroid metastasis from laryngeal cancer is extremely rare. Overall, thyroid gland involvement by metastatic carcinoma represents about 1.1–2.1 % among thyroid malignant diseases.

**Presentation of case:**

A male in his 70s came to our Otolaryngology Unit with a laryngeal squamous cell carcinoma (cT3). Total laryngectomy and bilateral neck dissection were performed. Histological examination revealed a pT3 carcinoma with sub-massive metastasis of the Delphian node. The patient underwent close follow-up. After eight months, neck examination revealed a suspected nodule in the right thyroid lobe. A right thyroid lobectomy was performed, and histological assessment revealed a nodule with squamous carcinoma metastasis in the superior pole of the thyroid lobe. The remaining thyroid tissue was affected by multinodular macrofollicular goitre. The patient underwent adjuvant therapy. One year after the second surgery, he showed no signs of recurrence.

**Discussion:**

Thyroid gland metastasis from laryngeal carcinoma is a very rare occurrence. In literature, we found only three articles that describes thyroid metastasis in overall 7 patients affected by laryngeal squamous cell carcinoma. Positive Delphian lymph node is usually related to poor prognosis: in 2007 a study reported tumour recurrence in 15 out of 25 patients with metastatic Delphian lymph node within the first two years of surgery.

**Conclusion:**

Thyroid gland metastasis from laryngeal carcinoma is rare; so close follow-up of oncologic patients is mandatory and, most of all, the positive Delphian node should not be underestimated for its predictive value.

## Highlights

• Thyroid metastases from laryngeal squamous cell are extremely rare;
• Thyroid metastases could occur through blood or lymphatic dissemination or direct spreading from adjacent organs;
• Positive Delphian lymph node is related to poor prognosis with risk of tumour recurrence within the first two years of surgery.

## 1 Introduction

Laryngeal carcinoma represents the 22nd most common cancer worldwide according to Global Cancer Statistics 2020 with 184.615 new cases and 99.840 deaths [1]. Squamous cell carcinoma represents the most common histotype with about 95 % of cases. First performed by Billroth in 1873, total laryngectomy is today limited only to advanced stages of laryngeal cancer (some T3 and T4a stages) where preservation surgery is not feasible or failed [2]. According to NCCN (National Comprehensive Cancer Network) Guidelines (version 1.2023), total laryngectomy surgery should be performed within ipsilateral or bilateral neck dissection and possible central compartment nodes dissection, with or without thyroidectomy, also in case of cN0 [3]. Thyroid metastasis from laryngeal cancer is extremely rare. Overall, thyroid gland involvement by metastatic carcinoma represents about 1.1–2.1 % among thyroid malignant diseases [4]. The most common primary tumour sites are lung, breast, and kidney [4,5].

We report the case of a male patient in his seventies who developed a thyroid metastasis 8 months after total laryngectomy and bilateral neck dissection surgery due to laryngeal cancer pT3. This work was written according to the SCARE criteria [6].

## 2 Case report

A male in his 70s with worsening dysphonia came to out Ear, Nose & Throat Unit. He had history of smoking for 50 years of about 30–40 cigarettes per day. Negative history for alcohol abuse and comorbidities.

Flexible laryngoscopy showed an exophytic lesion that affected the left hemilarynx, absence of arytenoid motility with subglottic, anterior commissure and right vocal cord extension. Neck computed tomography (CT) with contrast showed thickening of the left vocal cord with inhomogeneous enhancement, spreading in left paraglottic space with no extension in subglottis; increased in size and multinodular thyroid gland; no evidence of thyroid cartilage invasion and neck lymphadenopathy. The endoscopic biopsy of the laryngeal lesion revealed a squamous cell carcinoma. So, after discussing the case with a multidisciplinary team, a total laryngectomy within bilateral neck dissection (levels IIA–IIB–III–IV–V–VI) and tracheo-esophageal puncture (TEP) with voice prosthesis placement were performed. No complications occurred. During hospitalization, in 10th post operative day, naso-gastric tube was removed, and voice rehabilitation has been started. Twenty days after surgery patient was discharged at home. Histological examination revealed a pT3 poorly differentiated squamous cell carcinoma, free resection margins, no thyroid cartilage involvement and absence of metastasis on bilateral lymph nodes. Also, thyroid pyramidal lobe was free of cancer. However, in a 7 mm Delphian node a sub-massive metastasis was found. So, in agreement with oncologist, patient underwent close follow-up including neck CTs every three months.

**Fig. 1:**
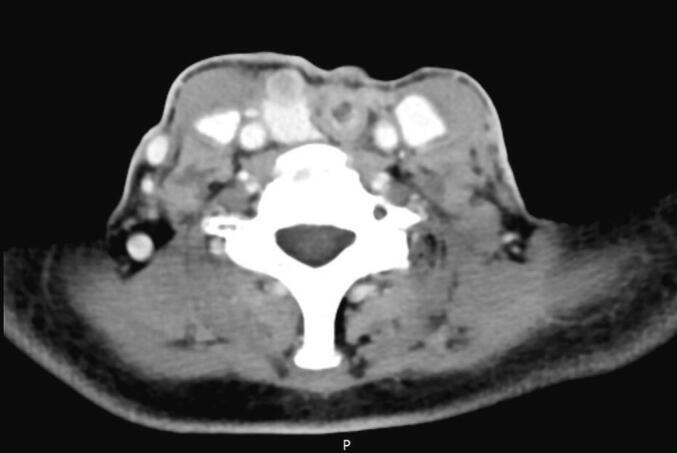
Axial neck CT scan with contrast: round extra-parenchymal lesion with peripheral contrast in the superior pole of the right thyroid lobe.

However, eight months after surgery, during follow-up control, neck examination revealed a suspected nodule in the right thyroid lobe. Neck CT with contrast showed a round extra-parenchymal lesion with peripheral contrast in the superior pole of the right thyroid lobe (Fig. 1).

So, a right thyroid lobectomy was performed under general anesthesia. Histological assessment revealed a nodule, 22 mm in diameter, in the superior pole of the thyroid lobe, that was a metastasis from squamous carcinoma. The remaining thyroid tissue was affected by multinodular macrofollicular goitre.

The patient underwent adjuvant therapy and, one year after the second surgery, the patient showed no clinical or radiological signs of recurrence.

## 3 Discussion

Laryngeal cancer usually leads to neck lymph node and lung involvement, as locoregional and distant metastases respectively. Thyroid gland metastasis from laryngeal carcinoma is a very rare occurrence. In literature, we found only three articles that describes thyroid metastasis in overall 7 patients affected by laryngeal squamous cell carcinoma [5,7,8]. However, in their studies, some authors excluded thyroid metastasis from laryngeal cancer due to possible direct extension from primary tumour [4,9]. Indeed, thyroid gland involvement from metastatic cancer could occur through blood or lymphatic dissemination or direct spreading from adjacent organs, like larynx [8,[10], [11], [12]]. In their systematic review, Kumar et al. proved that risk of thyroid involvement is greater in case of subglottic extension of the tumour [13]. Pasha et al. carried out a study about frequency of thyroid involvement due to laryngeal cancer and found that 12 out of 118 patients had thyroid gland invasion and that the pathways of spreading were subglottic tumour or erosion of thyroid cartilage (pT4) [10,14]. In our case, we can rule out a direct involvement of the thyroid gland; indeed, cancer involved only the left hemilarynx, thyroid cartilage was unaffected, and pyramidal lobe was tumour free. Moreover, as shown in CT image, in our case, the thyroid metastasis was extra-parenchymal: this proves that it wasn't a direct extension of primary tumour, but its involvement was due to transit metastasis. Metastatic Delphian lymph node was found. Positive Delphian lymph node is usually related to poor prognosis: in 2007 a study reported tumour recurrence in 15 out of 25 patients with metastatic Delphian lymph node within the first two years of surgery [15]. As well as described by this study, in our case, thyroid metastasis was detected about 8 months after total laryngectomy.

According to the poor literature, thyroid gland metastasis usually occurs in elderly (60–70 years old). Patients usually present a single asymptomatic lump, usually on the right thyroid lobe [5,9], exactly as occurred in our patient.

Moreover, as reported by Mistelou et al., thyroid metastases may occur concurrently with other thyroid lesions, like multinodular goitre and Hashimoto's thyroiditis [5], as well as in our case where multinodular macrofollicular goitre was found. A multinodular goitre or benign thyroid nodule may be responsible for a misdiagnosis or for a delay in diagnosis of thyroid metastasis.

## 4 Conclusion

Thyroid metastases from laryngeal squamous cell carcinoma – except in cases of direct spreading – are very rare but may occur. So, careful and close follow-up of oncologic patients is mandatory and, most of all, the positive Delphian lymph node should not be underestimated, precisely because it's prophetic as Apollo in the temple of Delphi.

## Ethical approval

We acquired the consent for publication from patient but we don’t require ethical approval since it is a anonymous case report.

## Funding

This research did not receive any specific grant from funding agencies in the public, commercial, or not-for-profit sectors.

## Author contribution

Carmelo Saraniti: concept of design, writing the paper, validation of final version

Barbara Verro: data collection and analysis, writing the paper

Antonio Lo Casto: data analysis and validation of the final version

## Guarantor

Carmelo Saraniti

## Note

Written informed consent was obtained from the patient for publication of this case report.

## Declaration of competing interest

The authors declare no conflict of interest
